# Bird Richness and Abundance in Response to Urban Form in a Latin American City: Valdivia, Chile as a Case Study

**DOI:** 10.1371/journal.pone.0138120

**Published:** 2015-09-30

**Authors:** Carmen Paz Silva, Cristóbal E. García, Sergio A. Estay, Olga Barbosa

**Affiliations:** 1 Instituto de Ciencias Ambientales y Evolutivas Universidad Austral de Chile, Valdivia, Chile; 2 Center for Applied Ecology and Sustainability (CAPES), Santiago, Chile; 3 Instituto de Ecología y Biodiversidad (IEB), Santiago, Chile; 4 Centro de Desarrollo Urbano Sustentable (CEDEUS), Santiago, Chile; University of Sydney, AUSTRALIA

## Abstract

There is mounting evidence that urban areas influence biodiversity. Generalizations however require that multiple urban areas on multiple continents be examined. Here we evaluated the role of urban areas on avian diversity for a South American city, allowing us to examine the effects of urban features common worldwide, using the city of Valdivia, Chile as case study. We assessed the number of birds and their relative abundance in 152 grid cells of equal size (250 m^2^) distributed across the city. We estimated nine independent variables: land cover diversity (DC), building density (BD), impervious surface (IS),municipal green space (MG),non-municipal green space (NG), domestic garden space (DG), distance to the periphery (DP), social welfare index (SW), and vegetation diversity (RV). Impervious surface represent 41.8% of the study area, while municipal green, non-municipal green and domestic garden represent 11.6%, 23.6% and 16% of the non- man made surface. Exotic vegetation species represent 74.6% of the total species identified across the city. We found 32 bird species, all native with the exception of House Sparrow and Rock Pigeon. The most common species were House Sparrow and Chilean Swallow. Total bird richness responds negatively to IS and MG, while native bird richness responds positively to NG and negatively to BD, IS DG and, RV. Total abundance increase in areas with higher values of DC and BD, and decrease in areas of higher values of IS, SW and VR. Native bird abundance responds positively to NG and negatively to BD, IS MG, DG and RV. Our results suggest that not all the general patterns described in previous studies, conducted mainly in the USA, Europe, and Australia, can be applied to Latin American cities, having important implications for urban planning. Conservation efforts should focus on non-municipal areas, which harbor higher bird diversity, while municipal green areas need to be improved to include elements that can enhance habitat quality for birds and other species. These findings are relevant for urban planning in where both types of green space need to be considered, especially non-municipal green areas, which includes wetlands, today critically threatened by urban development.

## Introduction

Urbanization is one of the most extreme forms of habitat transformation and it is increasing at a high rate [1]. The transformation of natural habitats into agricultural and urbanized areas results in a mosaic of land types ranging from heavily built urban centers to natural or semi-natural areas [2]. These extensive modifications have profound effects on the structure and function of ecosystems and are strongly correlated with adverse impacts on avian native communities [3, 4]

While urbanization affects habitat structure and bird populations, recent research finds both a positive and negative influence of urbanization on bird species richness and abundance. The most recurrent pattern described for urban avifaunal distribution is a negative relationship between species richness and urbanization [5–8]. Species richness was predicted to decline as a result of the loss of natural habitat and the reduction of resource availability [9, 10]. However, other studies found bird richness increased with increasing and intermediate levels of urbanization [11]. The intermediate responses—maximum richness at intermediate levels of urbanization—were related to higher habitat heterogeneity (different land uses) and resource availability, such as food, water, predator refuge, and nesting sites [12–14]. Likewise, total bird abundance has also been found to increase with the level of urbanization [10, 15–19]. This response was attributed to a higher number of urban exploiter species [9, 14, 20–22].

Green areas have been recognized as important elements in cities with a positive influence on both people and avian populations. Green spaces in cities increase human well-being and quality of life [23–25] and provide a place for direct interaction with nature [24, 26]. Green areas may also improve neighborhood appearance and influence house prices [25]. Birds also benefit from urban green spaces. The proximity of large and small parks provide shelter, food and may also function as habitat corridors [10, 27, 28]. However, the existence of a green area by itself does not ensure higher species richness. Species richness depends also on the heterogeneity and structural complexity of the vegetation [29–31]. For example, the geographic origin of the vegetation is also important, as native flora may support more diverse bird communities [32, 33]. Thus, it is important to not only study the size of urban green spaces, but also the configuration, vegetation structure and management of the green space in order to better understand their influence on urban bird populations.

In addition to studying the effects of green space on avian populations, urban ecological research has found many links between socioeconomic patterns of a city and biological diversity in the region [33, 34]. For example, the distribution and heterogeneity of vegetation is better correlated with the socioeconomic status of residents [29, 35, 36] than for example to the urban rural gradient. These patterns are relevant as the avifaunal diversity is strongly associated with the structure, diversity and complexity of vegetation [10, 14, 34, 37,38].

With an urban population reaching 80%, Latin America is the one of world’s most urbanized human populations region [39]. However, very few studies about urban ecology, particularly related the influence of urbanization on bird populations, have been conducted in the region [40]. Latin America harbors some of the world’s most biodiversity-rich ecosystems and it is home to seven of the world´s 35 recognized hotspots [41]. On the other hand, economic growth of the region depends heavily on natural resource exploitation, and thus, high rates of environmental degradation and biodiversity loss are common [42]. Latin American cities are also characterized by extreme social and economic inequality that influences urban form and the potential biodiversity they can harbor [25,43]. These unique characteristics of Latin American cities provide an opportunity to generate new knowledge and thus broader ecological understanding of urban systems. A comprehensive literature review, covering the last four decades, makes clear the scarcity of urban ornithological research in Latin America [44]. The few studies that exist on this topic in Latin America found a negative association of bird richness with urban development and the inverse relationship for bird abundance [45–47], and higher abundance of exotic bird populations [45,48]. Despite these general results, neither these studies nor studies from other regions analyzed the effect of city layout and infrastructure on avian community structure (but see [49]).

**Fig 1:**
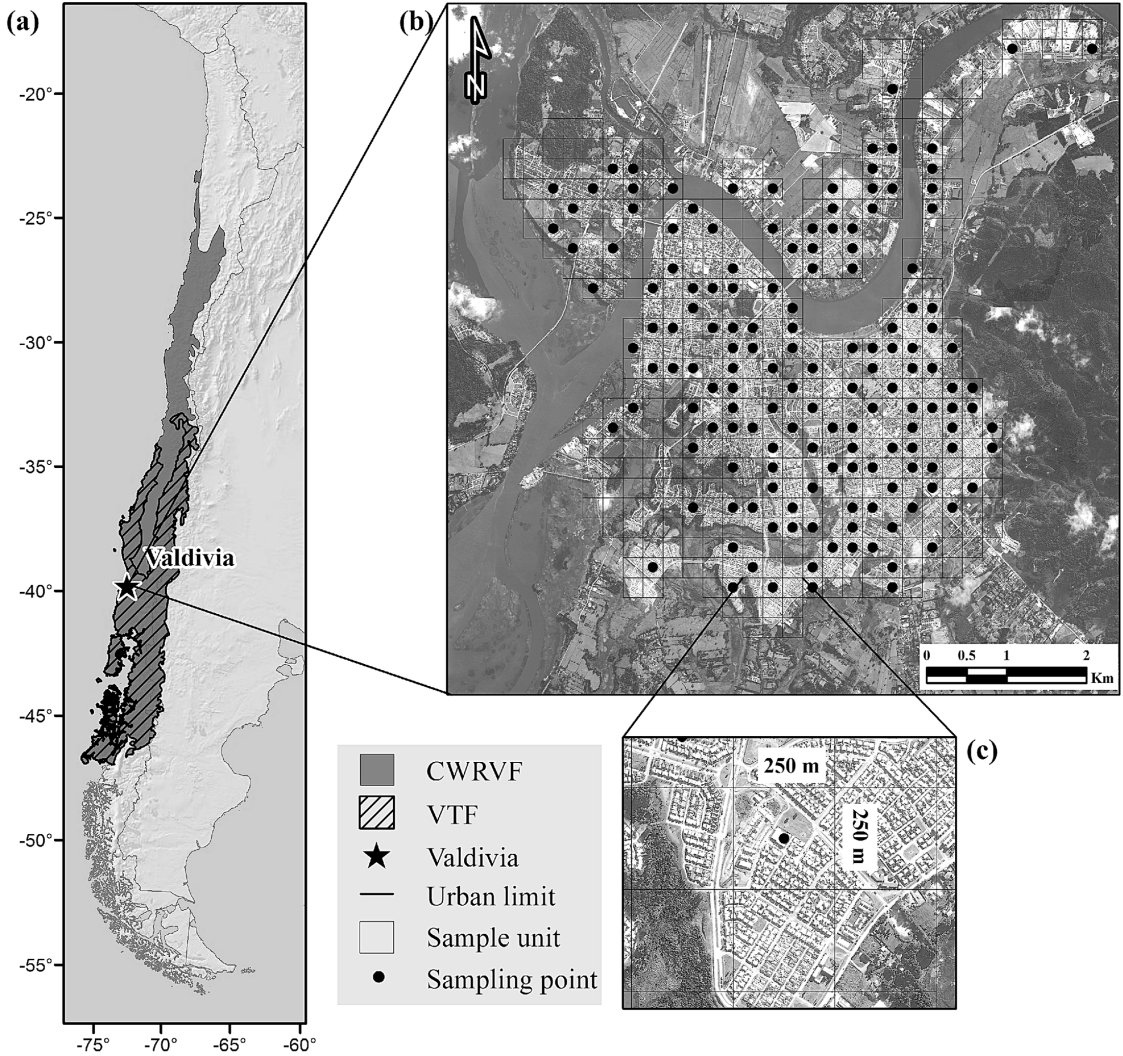
Study area. (a) Location of Valdivia in Chile. (b) Urban limits of Valdivia and the grid designed to select the sample units. Black dots indicate the sample units. (c) Detail of a sampling unit (250 m x 250 m cells). Abbreviations as follow: CWRVF, Chilean Winter Rainfall-Valdivian Forests; VTF, Valdivian Rain Forest ecoregion. Reprinted from [Silva CP, 2014] under a CC BY license, with permission from [Silva CP], original copyright [2014].

In this study, we explored how avian communities respond to urban form. Specifically, we examined how bird species richness and abundance relate to detailed components of urban form such as building density, impervious surface, green space, land cover diversity, vegetation richness and other factors such as the distance to the city’s periphery and socioeconomic status. We expect that both impervious and green space surface will affect avian distribution a city level scale, however, higher resolution scale variables, such as building density and green space characteristics, will have a stronger influence on bird richness and abundance. We used the city of Valdivia, in Southern Chile, as our case study (Fig 1). Valdivia is an ideal city for testing the effects of urbanization on bird populations as it is an intermediate fast growing city with rapidly changing features of urban form. In addition, it is located in the Chilean Winter Rainfall-Valdivian Forests biodiversity hotspot [41], and thus, a high priority area for conservation.

## Materials and Methods

### Ethics Statement

No specific permissions were required to conduct this work. All bird surveys were conducted in areas which are open to the public; therefore there was no need to ask land managers for approval. Research did not involve endangered or protected species or collection of biological material.

### Study area

The city of Valdivia, Chile (39° 48’ S, 73° 14’ W), is situated within the Valdivian Rain Forest ecoregion (Fig 1), and has a population of ca. 140,000 [50]. The landscape surrounding the urban area is predominantly silvo-agricultural, but there are also areas of well-preserved native forests [51, 52].

### Spatial analysis unit

We generated a spatially explicit database for the city in GIS, given that no previous digital database was freely available. Using the Fishnet application within ArcGis® 9.3 (ESRI) we created a grid with equal cells each 250 m x 250 m (6.25 ha) to ensure independence from urban form (Fig 1). The city’s administrative boundaries extend 42.39 km^2^ [53], but our study was restricted to predominantly urbanized areas with building cover ≥12.5% for residential and industrial areas inside the administrative borders of the city. This resulted in 434 grid cells and a total study area of 27 km^2^.

### Urban form

Seven of the nine variables used in this study, land cover heterogeneity, building density, impervious surface, municipal green space, non-municipal green space, domestic garden space, and distance to the urban limits, describe urban form. Data was extracted using both supervised classification and photointerpretation techniques.

We used a high definition digital image (Geoeye image 0.5 m2, multispectral, acquired in April 2010), with adequate resolution to detect urban form and green space differences among cells [25]. Land cover types were obtained through supervised classification using Maximum Likelihood (ML) as decision criterion (see [54, 55]), from a 2010 Geoeye-1 satellite pansharpened image (4 spectral bands and 0.5 m2 resolution). Classification was based on the 4 original spectral bands of the image. To highlight the differences between cover types, we generated two additional bands performing a principal component analysis (PCA) with the original bands and calculating the Normalized Divergence Vegetation Index (NDVI), as in Lillesand et al (2007) [56]. Tree cover was identified using discriminant criteria in zones with both high NDVI and high variance, in 7x7-window analysis as described in Zhang (2001) [57]. The resulting classification was re-sampled in a window analysis of 3x3 pixels to decrease the noise commonly derived from the use of pixel-based methods, like the ones used here [56]. These resulted in seven different land cover types divided by impervious and non-impervious surface categories: a) impervious surface (being one land cover type) and built surface, and b) non-impervious surface, including; bare soil, grassland, woodland, wetlands, and water, being the other five land cover types. We validated the above cover types in 200 random points generated within the limits if our study site, in which we compared the supervised classification data with field observations, using a confusion matrix, overall accuracy and kappa index [58].

We calculated land cover heterogeneity considering the seven land cover types (impervious surface, built surface, bare soil, grassland, woodland, wetlands, and water) resulting from the supervised classification, expressed through Simpson Index.

Additionally through photointerpretation, we digitalized each building and green space within the 434 grid cells of 250x250m. Buildings constructed after April 2010, where digitized using architectural plans from municipal council. This enabled us to quantify the building density, the number of buildings per cell, as a measure of urbanization. We also classified public green spaces as any parcel of permeable surface with open access to public use, such as municipal parks or plazas, public gardens, sports fields, cemeteries, and vacant lots. Vacant lots in Valdivia often include urban wetlands, with many having an unknown ownership status in the absence of a national inventory. Public green space types were defined as two types (municipal and non-municipal). Municipal green space was administrated by the municipality (municipal parks, plazas and cemetery gardens), and non-municipal spaces are those not under municipal administration (remaining spaces mentioned above). An additional category, domestic gardens, was calculated as all permeable space derived from the land cover data present in the residential city blocks, minus municipal and non-municipal green space area.

Distance to the periphery was calculated as the distance from the centroid of each cell to the closest urban limit.

### Socioeconomic index

We used the social welfare index as a proxy to evaluate socioeconomic status. This index was constructed using a combination of socioeconomic variables at the household level, such as house building materials, household employment and education level [59]. This value was assigned to each grid cell by a weighted average, adjusted by the area of each land cover type and averaged for the census output area.

### Vegetation richness

We evaluated plant species richness in every municipal and non-municipal green space present in the 152 cells previously selected for bird surveys (see bird surveys section). We calculated the proportion of green space inside each cell and surveyed plant species within every green space. Plant surveys were conducted during Spring and Summer 2011–2012. Every single plant present in each of the parcels was identified and classified considering both growth form (trees, shrubs and herbs) and geographic origin: native (to Chile), or exotic plants. Each parcel was georeferenced with a Garmin Etrex 30 GPS unit.

### Bird surveys

To assess avian response to urban form, we conducted bird surveys in 152 grid cells to evaluate bird species richness and relative abundance. We selected 152 cells from the total 434 using a stratified random sampling technique, representing a range of building density values to incorporate all levels of urbanization (see S1 Table; Fig 1). The centroid of each grid cell was used as the sampling point. Using a fixed 50-m radius point-count methodology [60, 61], we registered all birds seen or heard during 6 minutes. If the point was inaccessible via public right of way, the nearest accessible point within the same grid cell was chosen and recorded using a Garmin Etrex 30 GPS unit. Surveys were conducted during the breeding season of 2011 (September–December) within four hours after dawn. Each point was surveyed three times. The minimum distance between survey points was 250 m; adjacent cells were not surveyed during the same day to avoid overlapping observations of individual birds. Species abundance for each cell corresponded to the highest number of birds counted by species during three point surveys. We assumed that any bias associated with bird detectability was constant, because the number of bird species present in the city is small, with less than 45 possible species [62]. All species are easy to recognize and were surveyed at fairly close distance (50 m or less). Rarefaction curves were built to evaluate if the sampling effort was adequate (S1 Fig). Avian species richness was defined as the total number of species detected at each site during the study.

### Data analysis

We analyzed four response variables: total bird richness (BS), bird richness considering only native species (BSn), total bird abundance (BA) and bird abundance considering only the native species (BAn), and nine explanatory variables (Table 1): *land cover heterogeneity* (DC), *building density* (BD), *impervious surface* (IS), *municipal green space* (MG), *non-municipal green space* (NG), *domestic garden space* (DG), *distance to the urban limits* (DP), *social welfare index* (SW), and *vegetation richness* (RV).

**Table 1:** Variable abbreviation and descriptions.

Variable	Abbreviation	Variable description
Land cover diversity	DC	Simpson index expressed in land cover units.
Building density	BD	Residential and commercial buildings (b/ha).
Impervious surface	IS	Total area of manmade surface including roads and buildings (m^2^).
Municipal green space	MG	Total area of green space maintained by the city council (m^2^).
Non-municipal green space	NG	Total area of green space not maintained by the city council, including wetlands, private and unknown owner areas (m^2^).
Domestic garden space	DG	Total area of domestic gardens (m^2^).
Distance to periphery	DP	Distance from the centroid of each cell to the closest urban limit (m).
Welfare social index	SW	Socioeconomic status index.
Vegetation richness	RV	Number of plant species, including trees, shrubs and herbs.

Prior to analysis, the bird data were analyzed for autocorrelation by means of Moran’s index. We did not find significant spatial autocorrelation for species richness (Moran’s I = 0.018, p = 0.008). For species abundance, autocorrelation was significant but the Moran Index was sufficiently small to indicate that its effect would be negligible (Moran’s I = -0.008, p = 0.7). These results indicate that the numbers of bird species in cells close to each are not more similar than in cells situated farther apart.

To assess the effect that urban attributes have on bird richness and relative abundance, we applied two complementary analyses. First, we used generalized lineal models (GLM) with the Poisson error distribution and stepwise variable selection based on Akaike Information Criterion (AIC; [63]) to evaluate the relative importance of each of our explanatory variables on bird richness and relative abundance. We fitted a full model including all variables, and then a reduced model including just those variables selected in the stepwise procedure. Coefficients were beta standardized to facilitate comparisons among variables. Second, we fit generalized additive models (GAMs) to identify possible non-linear relationships among the predictor variables and our dependent variables. Though relationships of bird species richness and urbanization are well described in other portions of the world, we did not impose any a priori assumptions about the strength and direction of relationships in our system. Therefore, we used cubic splines and with the Poisson error distribution to identify potential nonlinear relationship between the response and explanatory variables that were not detected by the GLM approach [64]. The form of the partial functions related to each variable was determined by fitting cubic regression splines to the data. The complexity of the curve (the number of degrees of freedom) and the smoothing terms were determined by penalized regression splines and generalized cross validation (GCV) to avoid overfitting [64]. To allow variable selection (equivalent to the stepwise procedure in the GLM), we used cubic regression splines with shrinkage [64]. All statistical analyses were performed in R [65] using stats, mgcv, version 1.7–22; [64] and vegan libraries, version 2.0–7, [66]. Finally we evaluated multicollinearity using the variance inflation factor (VIF). VIF values were much lower than the suggested threshold (>10; [67]) in all models (highest observed value was 2.9).

## Results

### Urban form

Building density ranged from 0 to 391 buildings (b) per cell (0–0.62 b/ha). The proportion of total impervious surface was 41.8% of the total study area. Building density was positively correlated with impervious surface (r = 0.7, p<0.01) and negatively related with total green space (r = −032, p<0.01). Of the total non-manmade surface (1563.2 ha), 11.6% corresponded to municipal green space and 23.6% to non-municipal green space. The distribution of the land cover types in these areas differ notably. Tree cover represents only 31% of the land cover type in the municipal green, while it reaches 51% in non-municipal green. Unvegetated areas inside green spaces are relatively common. Some include hard surfaces which are impervious (paths, terraces, or other landscaping features that require less maintenance than grassland), and others, include bare soil mainly under playgrounds or sections with poor maintenance. Unvegetated and bare soil land cover types represent 64% and 32% of the total area of the municipal and non-municipal green, respectively. Water bodies represent only 3% of the land cover types in municipal green, while it represents 17% in non-municipal green. Wetlands represent 1.7% and 70.3% of the municipal and non-municipal green space respectively. Garden space contributes to 16% of the total urban area, with an average size of 101 m^2^. Although we did not evaluate land cover types inside private gardens, they were calculated from the category of non-impervious surface in residential areas and likely include a combination of bare soil, woods, grass, wetlands and water land cover types.

### Species richness, abundance and urban form

We identified 339 plant species. Only 99 (25.4%) of these species were identified as native. Exotic species richness was higher for the three growth form category (Table 2).

**Table 2:** Number of plant species by growth form at observation points.

Growth form	Native	Exotic	Total
Trees	44 (28.2%)	112 (71.8%)	156
Shrubs	31 (19.9%)	97 (62.2%)	128
Herbs	24 (15.4%)	91 (58.3%)	115
**Total**	99 (24.8%)	300 (75.2%)	399

Percentage in relation to the total number of species is shown in parenthesis.

We found thirty-two (32) bird species from an equal number of different genera (S2 Table). With the exception of House Sparrow (*Passer domesticus*) and Rock Pigeon (*Columba livia*), all observed species are native to Chile, including two endemic species, the Chilean Mockingbird (*Mimus thenca*) and the Chilean Tinamou (*Nothoprocta perdicaria*). Total observed species richness ranged from one to ten species per grid cell (mean = 3.77, coefficient of variation = 44.82%). While no species was found at every site, the most common species, the House Sparrow and Chilean Swallow (*Tachycineta meyeni*), were detected in 93% and 69% of the cells, respectively.

**Fig 2:**
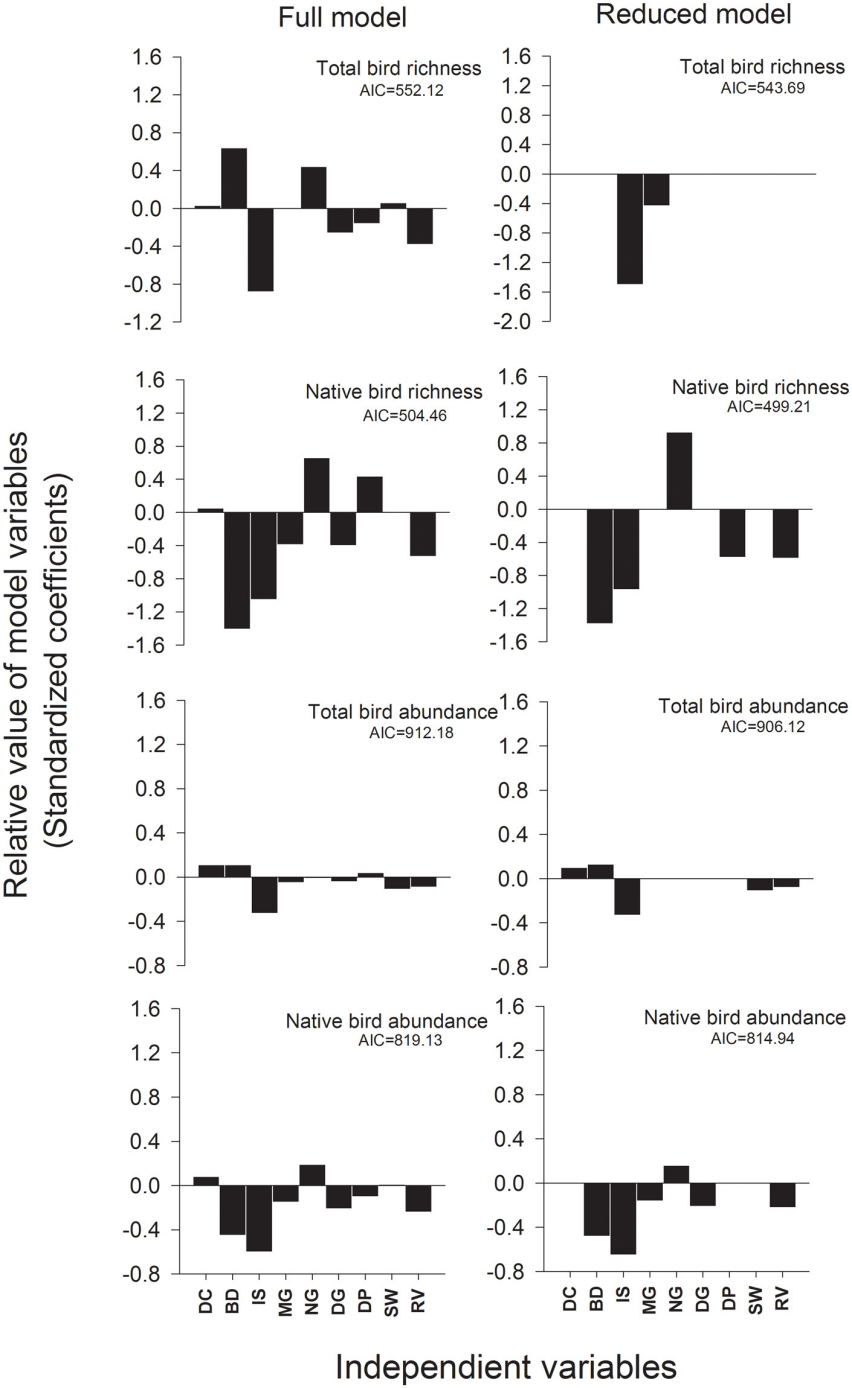
Relative importance of variables on the GLM models. Both full model (including all variables) and the reduced model (according to the stepwise reduction) are shown. AIC is the Akaike Information Criteria for the model. Coefficients are standardized to evaluate the relative importance of each variable in the model. Abbreviations as follow:, DC land cover diversity, BD building density, IS impervious surface, MG municipal green space, NG non-municipal green space, DG domestic garden space, DP distance to the periphery, SW social welfare index and RV vegetation richness.

The reduced GLM model for total bird richness only retained *municipal green*, and *impervious surface* as important variables (Fig 2, details in S3 Table), both with a negative influence on bird richness (MG = − 0.043, IS = − 0.150). These variables explain a fair amount of the null deviance (≈ 37%). In contrast, the reduced GLM model for native bird richness contains four variables that were negatively associated with native bird richness: *building density*, *impervious surface*, *vegetation richness* and *distance to the periphery* (BD = − 0.138; IS = − 0.097; RV = − 0.059; DP = −0.058), and one variable *non-municipal green space* that showed a positive association (NG = 0.093). This model explains almost half of the null deviance (48%).

The reduced GLM for relative bird abundance retained five of the explanatory variables. Three of them, *impervious surface*, *vegetation richness* and *welfare social index* showed a negative impact on relative bird abundance (IS = −0.033; RV = −0.008; SW = −0.011), while *building density and land cover diversity* had positive effects (BD = 0.013; DC = 0.010). Nevertheless, the explanatory power of this model was low (16%). Native bird abundance was explained in the reduced GLM model by six variables; five of them show a negative influence; *building density*, *municipal green space*, *domestic garden space*, *impervious surface* and *vegetation richness* (BD = −0.048; MG = −0.016; DG = −0.021; IS = −0.065; RV = −0.022), and one, *non-municipal green space* was positively associated (NG = 0.016). The explained deviance for this model was 40.7%.

Variables that best describe bird richness and abundance are not the same, but when considering only native species the reduced models coincide in four out of six variables, *building density*, *impervious surface*, *non-municipal green space*, and *vegetation richness* (Fig 2).

For total and native bird richness GAMs suggested no nonlinearities between variables. In both cases, the reduced models showed similar explained deviance (38.4% and 44.9% respectively) compared with the respective reduced GLM models (36.8% and 48% respectively).

The reduced GAM model for total bird abundance retained six variables *building density*, *municipal green space*, *domestic garden space*, *impervious surface*, *vegetation richness* and *welfare social index* (Table 3). For native bird abundance all variables, except for *land cover diversity*, were retained. In both cases, GAM models explained a higher proportion of the deviance (48.2% and 52.2%) than their respective GLMs (16.1% and 40.7% respectively), which strongly suggest nonlinearities between variables. All variables in the reduced models for total bird abundance and five in the case of native bird abundance (*non-municipal green space*, *domestic garden space*, *impervious surface*, *social welfare index*, and *distance to the periphery*) show a clear non-linear relationship. The non-linear relationships are highlighted by the high values of the effective degrees of freedom (Table 3), however most of them are monotonic decreasing or increasing functions. The number of individuals decreases with *domestic garden space* along a steep slope until it reaches a threshold around 9.000 m2 of garden area (S2 Fig), below which the influence of the variable becomes close to zero. The same situation is observed in the case of native bird abundance, but the threshold occurs around the 8.000 m^2^ of *domestic garden space* (S3 Fig).

**Table 3:** Results of the GAM fitting for total bird richness (BS), native bird richness (BSn), total bird abundance (BA), and native bird abundance (BAn).

GAM	Model	AIC	D^2^
**Bird Richness (total)**			
Full model	Ln(BS) = 1.289 + s_1_(**DC**, df≈ 0) + s_2_(**BD**, df = 0.522) + s_3_(**IS**, df = 1.045)+ s_4_(**MG**, df = 0.692) + s_5_(**NG**, df = 0.105) + S_6_(**DG**, df = 0.256) + s_7_(**DP**, df ≈ 0) + s_8_(**SW**, df≈ 0) + s_9_(**RV**, df≈ 0)	543.32	38.4
Best Model	Ln(BS) = 1.289 + s_1_(**BD**, df = 0.522) + s_2_(**IS**, df = 1.045) +s_3_(**MG**, df = 0.692) + s_4_(**NG**, df = 0.105)+ s_5_(**DG**, df = 0.256)	543.31	38.4
**Bird richness (native)**			
Full model	Ln(BSn) = 0.857 + s_1_(**DC**, df≈ 0) + s_2_(**BD**, df = 0.850) + s_3_(**IS**, df = 1.047) + s_4_(**MG**, df = 0.795) + s_5_(**NG**, df ≈ 0) + s_6_(**DG**, df = 0.730) + s_7_(**DP**, df≈ 0) + s_8_(**SW**, df ≈ 0) + s_9_(**RV**, df ≈ 0)	498.77	44.9
Best Model	Ln(BSn) = 0.857+ s_1_(**BD**, df = 0.850) + s_2_(**IS**, df = 1.047) + s_3_(**MG**, df = 0.795) + s_4_(**DG**, df = 0.730)	498.76	44.9
**Bird abundance (total)**			
Full model	Ln(BA) = 2.436 + s_1_(**DC**, df = 5.846) + s_2_(**BD**, df = 3.14) + s_3_(**IS**, df = 9.972) + s_4_(**MG**, df = 13.484) + s_5_(**NG**, df = 11.010) + s_6_(**DG**, df = 7.340) + s_7_(**DP**, df = 3.61) + s_8_(**SW**, df = 5.731)+ s_9_(**RV**, df = 7.923)	887.79	64.7
Best Model	Ln(BA) = 2.442 + s_1_(**BD**, df = 2.840) + s_2_(**IS**, df = 9.972) +s_3_(**MG**, df = 12.338) + s_4_(**DG**, df = 6.439)+ s5(SW, df = 5.731) + s6(RV, df = 7.923)	875.87	48.2
**Bird abundance (native)**			
Full model	Ln(BAn) = 1.484 + s_1_(**DC**, df = 2.638) + s_2_(**BD**, df = 1.022) + s_3_(**IS**, df = 1.154) + s_4_(**MG**, df = 0.458)+ s_5_(**NG**, df = 2.234)+ s_6_(**DG**, df = 5.705) + s_7_(**DP**, df = 7.061) + s_8_(**SW**, df = 4.521)	785.98	52.4
Best Model	Ln(BAn) = 1.481+ s_1_(**BD**, df = 1.116) + s_2_ (**IS**, df = 2.845) + s_3_(**MG**, df = 0.702) + s_4_(**NG**, df = 2.215)+ s_5_(**DG**, df = 5.709) + s_6_(**DP**, df = 5.420) + s_7_(**SW**, df = 4.477) + s_8_(**RV**, df = 0.847)	785.66	52.2

Both full model (including all predictors) and the best model according to the shrinkage procedure are shown. s_i_ represents the cubic regression spline for the variable and *df* is the effective degrees of freedom of each term. AIC is the Akaike Information Criteria for the model and *D* ^2^ is the percentage of explained deviance.

Abbreviations as follow: DC land cover diversity; BD building density, IS impervious surface, MG municipal green space, NG non-municipal green space, DG domestic garden space, DP distance to the periphery, SW social welfare index, and RV vegetation richness.

## Discussion

Overall we found conspicuous relationships between the components of urban form and bird richness and abundance. Our findings are similar to general patterns described for urban birds in other regions of the world; however with high-resolution urban form data, we were able to detect patterns that have not been previously described (but see [31,68]). Here we show the importance of non-municipal green spaces on supporting bird diversity, by both their characteristics and their spatial location in city. The later, non–municipal green space, which doubles the amount of municipal green space, located in both affluent an less affluent areas of the city, buffer the influence of other variables such as socioeconomic status on bird richness and abundance.

In Valdivia, urban form variables related to bird species richness differed from those that affect bird abundance. As in other cities, building density and impervious surface were a negative influence on bird richness [11,69], while socioeconomic status had a positive, although weak relationship with bird richness [33,34,49]. On the other hand, total bird abundance, including both native and non-native species, was positively related to building density and negatively correlated to high socioeconomic status. Similar to bird species richness however, the abundance of native birds was positively related to socioeconomic status. That is, less affluent neighborhoods had a higher abundance of exotic birds, while more affluent areas had a higher abundance of native birds.

We found a negative, although weak, association of total and native bird abundance and richness with distance to the periphery of the city. Consistent with previous studies, this indicates that as the distance from the surrounding native forest and wetlands increases (near the city center) the number of individuals and species decreases [12,19,70]. The weak effect of distance to the periphery effect upon total bird abundance also matches with the findings of these previous studies. In Valdivia, as in other cities, this can be explained by the higher number of the two exotic species, the House Sparrow and the Rock Pigeon, but also the Chilean Swift (*T*. *meyeni*), a native species.

Although green spaces are generally thought to have a positive effect on bird diversity (e.g. [71–73]), our findings highlight that different categories of green space can have very different effects even exerting a negative influence, as is the case with municipal green areas. Our data show that municipal green spaces are more homogeneous with respect to land cover and vertical heterogeneity when compared to non-municipal green areas, as the first are designed and maintained for recreational purposes [74]. On the other hand, non-municipal green areas in Valdivia present higher structural complexity, offering a greater variety of habitats and food resources to birds. The structural heterogeneity is reflected in our results by higher bird richness in the non-municipal green spaces than the more highly managed, homogeneous municipal green spaces. Our study strongly suggests that less managed non-municipal green areas are important for biodiversity. Non-municipal green spaces—a unique feature of many Latin American cities—may be particularly important for preserving native bird diversity in quickly urbanizing areas of Latin America. Latin American cities have a pattern of growth with little or no urban planning, expanding with a discontinuous and scattered pattern that generates these interstitial areas as a product of the consolidation of informal settlements in the urgent need to provide housing [39]. More interestingly, in Valdivia these areas include a vast proportion of wetlands (70.4%), which not only have been prone to transformation due to high development pressure [75], but are also neglected and being used as dumping sites [76].

Surprisingly, bird richness and abundance were not related to vegetation richness in our study region. This result is contrary to many other studies (i.e. [32,73,77]). The lack of this effect may be attributed to the generally high abundance of vegetation, and high number of exotic vegetation species (75.2% of total in this study) in Valdivia urban limits. Previous studies reported that higher proportion of exotic vegetation supports a lower richness and abundance of bird species [32].

Several studies have addressed the importance of domestic gardens for supporting higher biodiversity (e.g. [78–84]), but our results are dramatically different, suggesting interesting differences with reported patterns for cities in the developed world cities. The individual size and total area of domestic gardens in Valdivia could explain the negative relationship between bird richness, abundance and garden space. In Valdivia, average domestic garden space is 101 m^2^ and overall represent only 16% of land area, which are lower than reported in studies that show a positive influence of domestic green areas for urban bird communities (see [78]).

Our findings provide essential information for urban planners and conservationists on the importance of key urban attributes, including urban density, impervious surfaces, and the management of green areas, for urban bird populations. Despite rapid urbanization, key features of the urban environment can be used to support the conservation of native bird populations, which is particularly important in biodiversity hotspots, such as Valdivia Chile. In future urban development plans, we recommend that special attention be paid to: 1) municipal green areas, where the habitat quality for birds can be improved through the reduction of the impervious surface and the creation and conservation of multi-layered vegetation structure, and 2) the preservation of non-municipal green areas, including the wetlands that are critically threatened by urban development in Valdivia.

## Supporting Information

S1 Fig Rarefaction curves showing the expected number of bird species for any given number of sites. Bird survey #1 (blue), bird survey #2 (orange), bird survey #3 green. (TIFF)

S2 Fig Smoothed relationships between total bird abundance and urban form variables. Smoothed relationships between total bird abundance (BA) and urban form variables, DC land cover diversity, BD building density, IS impervious surface, MG municipal green space, NG non- municipal green space, DG garden space, DP distance to the periphery, SW social welfare index, and RV vegetation richness.
(TIFF)

S3 Fig Smoothed relationships between native bird abundance and urban form variables. Smoothed relationships between native bird abundance (BAn) and urban form variables, DC land cover diversity, BD building density, IS impervious surface, MG municipal green space, NG non- municipal green space, DG garden space, DP distance to the periphery, SW social welfare index, and RV vegetation richness.
(TIFF)

S1 Table Categories of building densities (DOCX)

S2 Table Observed species list. (DOCX)

S3 Table Results for the GLM fitting for total bird richness (BS), native bird richness (BSn), total bird abundance (BA) and native bird abundance (BAn). (DOCX)

S1 Text Fig 1 permission. (DOCX)
