# Supplementary figures and images for: Bird Richness and Abundance in Response to Urban Form in a Latin American City: Valdivia, Chile as a Case Study
Source: PLoS One. 2015 Sep 30;10(9):e0138120. doi: 10.1371/journal.pone.0138120 (PMC4589359; doi:10.1371/journal.pone.0138120)

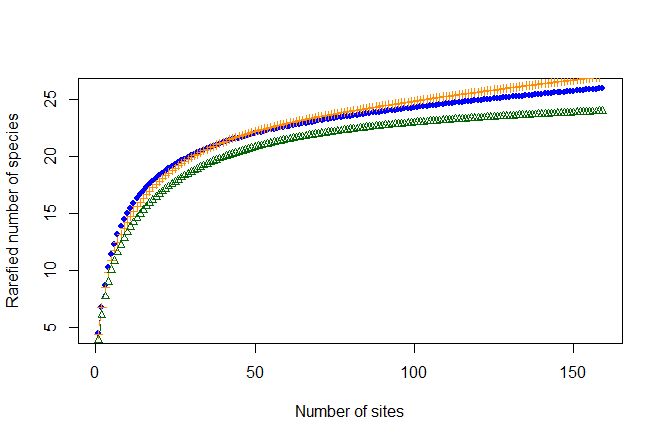

Supplement: S1 Fig — (TIFF) [file pone.0138120.s001.tiff]

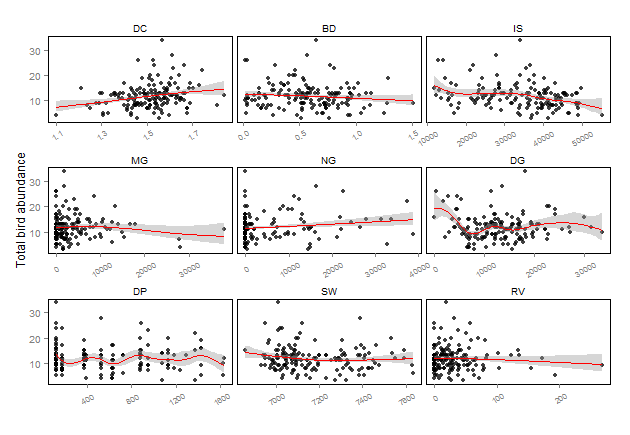

Supplement: S2 Fig — Smoothed relationships between total bird abundance (BA) and urban form variables, DC land cover diversity, BD building density, IS impervious surface, MG municipal green space, NG non- municipal green space, DG garden space, DP distance to the periphery, SW social welfare index, and RV vegetation richness. (TIFF) [file pone.0138120.s002.tiff]

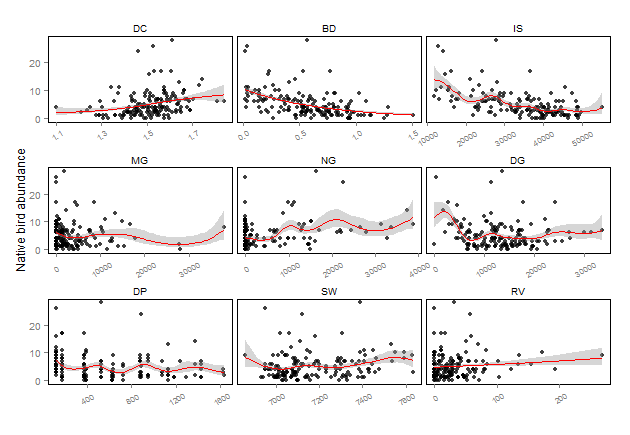

Supplement: S3 Fig — Smoothed relationships between native bird abundance (BAn) and urban form variables, DC land cover diversity, BD building density, IS impervious surface, MG municipal green space, NG non- municipal green space, DG garden space, DP distance to the periphery, SW social welfare index, and RV vegetation richness. (TIFF) [file pone.0138120.s003.tiff]
