# Supplementary material for: Bird Richness and Abundance in Response to Urban Form in a Latin American City: Valdivia, Chile as a Case Study
Source: PLoS One. 2015 Sep 30;10(9):e0138120. doi: 10.1371/journal.pone.0138120 (PMC4589359; doi:10.1371/journal.pone.0138120)
Supplement: S1 Table — (DOCX) [file pone.0138120.s004.docx]

| **Category** | **Number of buildings** | **Number of cells in the category** | **Number of cells surveyed in the category** |
| --- | --- | --- | --- |
| 1 | 04-53 | 51 | 27 |
| 2 | 54-103 | 67 | 34 |
| 3 | 104-152 | 72 | 40 |
| 4 | 153-201 | 50 | 31 |
| 5 | 202-250 | 23 | 14 |
| 6 | 251-300 | 6 | 4 |
| 7 | 301-349 | 3 | 2 |

**S1 Table. Categories for building densities and number of cells surveyed in each category**
