# Supplementary material for: Bird Richness and Abundance in Response to Urban Form in a Latin American City: Valdivia, Chile as a Case Study
Source: PLoS One. 2015 Sep 30;10(9):e0138120. doi: 10.1371/journal.pone.0138120 (PMC4589359; doi:10.1371/journal.pone.0138120)
Supplement: S2 Table — (DOCX) [file pone.0138120.s005.docx]

| Order | Scientific Name | Common names |
| --- | --- | --- |
| Cathartiformes | *Coragyps atratus* | Black Vulture |
|  |  |  |
| Charadriiformes | *Chroicocephalus maculipennis* | Brown-hooded Gull |
|  | *Vanellus chilensis* | Southern Lapwing |
|  |  |  |
| Columbiformes | *Columba livia** | Rock Pigeon |
|  | *Patagioenas araucana* | Chilean Pigeon |
|  |  |  |
| Falconiformes | *Falco sparverius* | American Kestrel |
|  | *Milvago chimango* | Chimango Caracara |
|  |  |  |
| Passeriformes | *Anairetes parulus* | Tufted Tit-tyrant |
|  | *Troglodytes aedon* | House Wren |
|  | *Cistothorus platensis* | Sedge Wren |
|  | *Zonotrichia capensis* | Rufous-collared Sparrow |
|  | *Sicalis luteola* | Grassland Yellow-finch |
|  | *Cinclodes patagonicus* | Dark-bellied Cinclodes |
|  | *Lessonia rufa* | Patagonian Negrito |
|  | *Xolmis pyrope* | Fire-eyed Diucon |
|  | *Elaenia Albiceps* | White-crested Elaenia |
|  | *Tachycineta meyeni* | Chilean Swallow |
|  | *Passer domesticus** | House Sparrow |
|  | *Sporagra barbata* | Black-chinned Siskin |
|  | *Sturnella loyca* | Long-tailed Meadowlark |
|  | *Molothrus bonariensis* | Shiny Cowbird |
|  | *Phytotoma rara* | Rufous-tailed Plantcutter |
|  | *Hymenops perspicillatus* | Spectacled Tyrant |
|  | *Mimus thenca* | Chilean Mockingbird |
|  | *Leptasthenura aegithaloides* | Plain-mantled Tit-spinetail |
|  | *Curaeus curaeus* | Austral Blackbird |
|  | *Turdus falcklandii* | Austral Thrush |
|  |  |  |
| Pelecaniformes | *Theristicus melanopis* | Black-faced Ibis |
|  |  |  |
| Suliformes | *Phalacrocorax brasilianus* | Neotropic Cormorant |
|  |  |  |
| Tinamiformes | *Nothoprocta perdicaria* | Chilean Tinamou |
|  |  |  |
| Trochiliformes | *Sephanoides sephaniodes* | Green-backed Firecrown |

**S2 Table.** **Observed species list**
