# Supplementary material for: Bird Richness and Abundance in Response to Urban Form in a Latin American City: Valdivia, Chile as a Case Study
Source: PLoS One. 2015 Sep 30;10(9):e0138120. doi: 10.1371/journal.pone.0138120 (PMC4589359; doi:10.1371/journal.pone.0138120)
Supplement: S3 Table — (DOCX) [file pone.0138120.s006.docx]

**S3 Table**. **Results for the GLM fitting for total bird richness (BS), native bird richness (BSn), total bird abundance (BA) and native bird abundance (BAn).**

| **GLM** | **Model** | **AIC** | **D^2^** |
| --- | --- | --- | --- |
| **Bird Richness (total)** |  |  |  |
| Full model | Ln(BS) = – 0.003 DC − 0.064 BD − 0.088 IS − 0.038 MG + 0.044 NG − 0.026 DG  − 0.016 DP + 0.006 SW− 0.038 RV | 552.12 | 42.2 |
| Best model (stepwise) | Ln(BS) = − 0.15 IS − 0.043 MG | 543.69 | 36.8 |
| **Bird richness (native)** |  |  |  |
| Full model | Ln(BSn) = 0.005 DC − 0.141 BD − 0.105 IS − 0.039 MG + 0.066 NG − 0.040 DG  + ≈0 SW − 0.044 DP − 0.053 RV | 504.46 | 48.4 |
| Best model (stepwise) | Ln(BSn) = − 0.138 BD − 0.097 IS + 0.093 NG − 0.058 DP − 0.059 RV | 499.21 | 48.0 |
| **Bird abundance (total)** |  |  |  |
| Full model | Ln(BA) = 0.011 DC − 0.011 BD − 0.033 IS − 0.005 MG − 0.001 NG − 0.004 DG  + 0.004 DP − 0.011 SW − 0.009 RV | 912.18 | 16.7 |
| Best model (stepwise) | Ln(BA) = 0.010 DC − 0.013 BD − 0.033 IS − 0.011SW + 0.008 RV | 906.12 | 16.1 |
| **Bird abundance (native)** |  |  |  |
| Full model | Ln(BAn) = 0.008 DC − 0.045 BD − 0.060 IS −0.015 MG + 0.019 NG −0.021 DG  − 0.010 DP + 0.001 SW − 0.024 RV | 819.13 | 41.1 |
| Best model (stepwise) | Ln(BAn) = − 0.048 BD − 0.065 IS − 0.016 MG + 0.016 NG − 0.021 DG  − 0.022 RV | 814.94 | 40.7 |

Both full model (including all variables) and the reduced model (according to the stepwise reduction) are shown. AIC is the Akaike information

criteria for the model and *D*^2^ is the percentage of explained deviance. Coefficients are standardized to evaluate the relative importance of each

variable in the model.

Abbreviations as follow: DC land cover diversity; BD building density, IS impervious surface, MG municipal green space, NG non-municipal green space, DG domestic garden space, DP distance to the periphery, SW social welfare index, and RV vegetation richness.
