# Supplementary material for: Bird Richness and Abundance in Response to Urban Form in a Latin American City: Valdivia, Chile as a Case Study
Source: PLoS One. 2015 Sep 30;10(9):e0138120. doi: 10.1371/journal.pone.0138120 (PMC4589359; doi:10.1371/journal.pone.0138120)
Supplement: S1 Text — (DOCX) [file pone.0138120.s007.docx]

**S1 Text Figure1 Permission**

Hereby I the author, Carmen Paz Silva, give permission to publish **Figure 1** under a CC BY license. Originally in

Silva CP (2014). Response of avian bird communities to urbanization in southern Chile: new emerging patterns for South America. Doctoral Dissertation, Universidad Austral de Chile, Chile.
